# A functional framework in patient fibroblasts informs *ATP7A* variant pathogenicity and identifies p.Q990P as a novel cause of distal motor neuropathy

**DOI:** 10.1093/hmg/ddag061

**Published:** 2026-07-08

**Authors:** Gonzalo Perez-Siles, Bianca R Grosz, Melina Ellis, Jonathan Baets, Ataf Sabir, Mary O’Driscoll, Rita Horvath, Kleopas A Kleopa, Sharon La Fontaine, Pontus Gourdon, Steve Vucic, Marina L Kennerson

**Affiliations:** School of Medical Sciences, Faculty of Medicine and Health, University of Sydney, Sydney, NSW 2050, Australia; Northcott Neuroscience Laboratory, ANZAC Research Institute, Gate 3 Hospital Road, Sydney Local Health District, Sydney, NSW 2139, Australia; School of Medical Sciences, Faculty of Medicine and Health, University of Sydney, Sydney, NSW 2050, Australia; Northcott Neuroscience Laboratory, ANZAC Research Institute, Gate 3 Hospital Road, Sydney Local Health District, Sydney, NSW 2139, Australia; Northcott Neuroscience Laboratory, ANZAC Research Institute, Gate 3 Hospital Road, Sydney Local Health District, Sydney, NSW 2139, Australia; Translational Neurosciences, Faculty of Medicine and Health Sciences, University of Antwerp, Campus Drie Eiken, Universiteitsplein 1, Antwerp 2610, Belgium; Laboratory of Neuromuscular Pathology, Institute Born-Bunge, University of Antwerp, Campus Drie Eiken, Universiteitsplein 1, Antwerp 2610, Belgium; Neuromuscular Reference Centre, Department of Neurology, Antwerp University Hospital, Drie Eikenstraat 655, Antwerp 2650, Belgium; West Midlands Regional Clinical Genetics Service and Birmingham Health Partners, Birmingham Women's and Children's Hospitals NHS Foundation Trust, Mindelsohn Way, Edgbaston, Birmingham B15 2TG, United Kingdom; Clinical Genetics Unit, Birmingham Women's and Children's NHS Trust, Mindelsohn Way, Edgbaston, Birmingham B15 2TG, United Kingdom; Department of Clinical Neurosciences, John Van Geest Centre for Brain Repair, School of Clinical Medicine, University of Cambridge, Robinson Way, Cambridge CB2 0PY, United Kingdom; Neuroscience Department, The Cyprus Institute of Neurology and Genetics, 6 International Airport Avenue, Ayios Dometios, Nicosia 2371, Cyprus; Center for Neuromuscular Disorders, The Cyprus Institute of Neurology and Genetics, 6 International Airport Avenue, Ayios Dometios, Nicosia 2371, Cyprus; School of Life and Environmental Sciences, Deakin University, 221 Burwood Highway, Burwood, VIC 3125, Australia; Department of Biomedical Sciences, Faculty of Health and Medical Sciences, University of Copenhagen, Blegdamsvej 3B, Copenhagen 2200, Denmark; Department of Experimental Medical Science, Faculty of Medicine, Lund University, Biomedical Centre (BMC I13), Sölvegatan 19, Lund SE-221 84, Sweden; Concord Clinical School, Faculty of Medicine and Health, University of Sydney and Brain and Nerve Research Centre, Concord Repatriation General Hospital, Hospital Road, Sydney, NSW 2139, Australia; School of Medical Sciences, Faculty of Medicine and Health, University of Sydney, Sydney, NSW 2050, Australia; Northcott Neuroscience Laboratory, ANZAC Research Institute, Gate 3 Hospital Road, Sydney Local Health District, Sydney, NSW 2139, Australia; Molecular Medicine Laboratory, Concord Repatriation General Hospital, Hospital Road, Sydney, NSW, 2139 Australia

**Keywords:** ATP7A, distal hereditary motor neuropathy, variant of unknown significance (VUS), fibroblasts, copper, trans-Golgi network, functional interpretation framework

## Abstract

ATP7A is a P-type ATPase copper transporter and a central component of the intracellular network to maintain copper (Cu) homeostasis, trafficking between the trans-Golgi network (TGN) and the cell periphery in response to intracellular Cu levels. Pathogenic *ATP7A* variants cause Menkes disease (MNK), occipital horn syndrome (OHS) and X-linked distal hereditary motor neuropathy (HMNX), with the clinical severity inversely related to residual ATP7A function. In MNK and OHS, variants result in loss of ATP7A function due to absent (MNK) or markedly reduced (OHS) protein levels, leading to abolished ATP7A trafficking and/or impaired Cu transport capacity. HMNX-associated variants are thought to retain partial Cu transport activity, although functional data remain limited. To date, three missense variants (p.T994I, p.P1386S, p.A991D) are established causes of HMNX, but the number of *ATP7A* variants reported in patients with a distal motor neuropathy phenotype is increasing, creating a need for functional assessment of variants of uncertain significance (VUS). We have developed a functional framework for evaluating HMNX-associated *ATP7A* variants. Using three patient fibroblast lines carrying the confirmed variants, we demonstrate reduced ATP7A TGN localization under low-Cu conditions and impaired capacity of these cell lines to maintain intracellular Cu levels. By applying these assays to five VUS (p.R703H, p.Y760C, p.A768G, p.Q990P, p.M1311V) identified in individuals from unsolved peripheral neuropathy families, we identify p.Q990P as a novel *ATP7A* variant in a patient with progressive peripheral motor neuropathy. This approach enables comparison of Cu trafficking and handling across ATP7A alleles, providing a functional framework to support diagnostic variant classification.

## Introduction

Cellular copper (Cu) homeostasis is tightly regulated by an integrated network of proteins that coordinate Cu uptake, intracellular distribution, utilization, and export. A central component of this network is ATP7A, a Cu-transporting P-type ATPase that plays a dual role in maintaining cellular Cu balance. Under basal Cu conditions, ATP7A predominantly localizes to the trans-Golgi network (TGN), where it supplies Cu to the secretory pathway for incorporation into newly synthesized cuproenzymes. In response to elevated intracellular Cu levels, ATP7A undergoes dynamic, regulated trafficking from the TGN to vesicular compartments and the plasma membrane thereby facilitating cellular Cu efflux and preventing toxic accumulation [1–3].

Variants in *ATP7A* cause three X-linked disorders: Menkes Disease (MNK - MIM #309400), occipital horn syndrome (OHS-MIM #304150) and X-linked distal motor neuropathy (HMNX-MIM #33489). The clinical severity of these inherited diseases inversely correlates with the residual function of the mutated ATP7A protein (for review [4]). Unless treated in the initial days after birth, MNK is a lethal neurodegenerative condition also characterised by connective tissue disturbances and hair and pigment abnormalities [5]. In MNK, Cu transport is irreversibly disrupted leading to profound copper deficiency in the blood, liver and brain [6]. In OHS patients (the milder form of MNK) connective tissue abnormalities are prominent and neurological symptoms are less profound, usually manifesting at 5 to 10 years of age [7]. Variants in *ATP7A* causing MNK and OHS, range from complete loss to severe loss-of-function of the Cu transporter resulting in graded deficits of protein activity and trafficking (for review [8]).

HMNX is a pure axonal distal motor neuropathy characterised by slowly progressive weakness and atrophy of distal muscles. Disease onset ranges from early childhood to late adult (2 to 50 years) and affected individuals typically show no or mild sensory involvement [9]. To date, three *ATP7A* missense variants (p.A991D, p.T994I and p.P1386S) have been reported from three unrelated HMNX families [9, 10]. Functional studies, limited to the initially reported T994I and P1386S variants [9] indicate that these alterations affect interactions of ATP7A with other proteins (p97/VCP [11] and the adaptor proteins AP-1 and AP-2 [12], respectively) resulting in subtle defects in ATP7A intracellular trafficking. Subsequent studies using complementary *in vitro* and *in vivo* models of the p.T994I variant confirmed a partial reduction in ATP7A protein levels and abnormalities in the subcellular localisation of the Cu transporter [13, 14].

Next generation sequencing technologies have rapidly increased the number of *ATP7A* variants identified in patients with motor neuropathy [15–18]. In cases where segregation studies are not feasible, there is a critical need for accessible functional assays to evaluate the pathogenicity of *ATP7A* variants of uncertain significance (VUS). In this study, we systematically assessed patient fibroblasts harboring the three known HMNX variants to define their effects on ATP7A protein levels, TGN localization under different copper-loading conditions and the capacity of cells to maintain intracellular copper levels following copper challenge. These experiments revealed a unifying molecular phenotype associated with pathogenic *ATP7A* variants in HMNX. We then used this molecular signature, to functionally characterise five *ATP7A* VUS (p.R703H, p.A768G, p.Y760C, p.Q990P, and p.M1311V). Based on initial *in silico* predictions and the complete absence of reported hemizygous variants in the gnomAD database (Table 1), p.A768G and p.Q990P are classified as variants of unknown significance (VUS) under ACMG criteria. In contrast, p.R703H, p.Y760C and p.M1311V are currently annotated benign to likely benign according to ACMG and ClinVar criteria. Using our functional framework in fibroblasts bearing these ATP7A variants, we validated a novel missense variant, (p.Q990P) as causative for a progressive peripheral motor neuropathy. Together, these findings further expand the genetic heterogeneity of HMNX.

**Table 1:** ATP7A variants assessed in this study and their in silico predictions.

**Patient number**	I	II	III	IV	V				
**Coding change**	c.2108G > A	c.2279A > G	c.2303C > G	c.2969A > C	c.3931A > G	c.2519A > T	c.2972C > A	c.2981C > T	c.4156C > T
**Protein Change**	p.R703H	p.Y760C	p.A768G	p.Q990P	p.M1311V	p.E840V	p.A991D	p.T994I	p.P1386S
**Genomic position (hg19)**	X:77267107	X:77268482	X:77268506	X:77284799	X:77298212	X:77271271	X:77284802	X:77284811	X:77300999
**Genomic position (hg38)**	X:78011610	X:78012985	X:78013009	X:78029302	X:78042714	X:78015774	X:78029305	X:78029314	X:78045502
**ClinVar Accession ID**	VCV000966195.8	VCV000533671.10	Unreported	Unreported	VCV000210466.50	VCV000465113.53	VCV000549669.1	VCV000011794.6	VCV000011795.13
**ClinVar Interpretation/s**	Uncertain significance (1); Likely benign (1)	Uncertain significance (2); Benign (1); Likely benign (1)	N/A	N/A	Uncertain significance (1); Benign (4); Likely benign (1)	Uncertain significance (1); Benign (2); Likely benign (1)	Likely Pathogenic	Pathogenic (1); Uncertain significance (1)	Pathogenic
**gnomAD (aggregated) v3**	37/1209005 (12 hemi)	22/1208816 (8 hemi)	3/1208798 (0 hemi)	0	626/1210029 (188 hemi)	202/1209903 (67 hemi)	0	0	0
**SIGMA**	0.045	0.445	0.197	0.967	0.049	0.174	0.875	0.496	0.390
**Revel**	0.17	0.90	0.88	0.85	0.66	0.82	0.79	0.92	0.92
**AlphaMissense**	0.057	0.801	0.384	0.965	0.189	0.715	0.969	0.960	0.963
**GERP**	−0.54	5.64	5.64	5.84	5.01	5.32	6.06	6.06	5.54
**EVE**	0.14	0.87	0.59	0.7	0.31	0.61	0.43	0.8	0.87
**SIFT**	0.341	0	0.005	0.002	0.009	0.001	0.015	0.001	0
**ACMG Classification**	Likely Benign (BS2, BP4)	VUS (BS2)	VUS (PM2, PP3)	VUS (PM1, PM2, PP3)	Benign (BA1)	Likely Benign (BS2, BP4)	VUS (PM1, PM2, PP3)	Likely Pathogenic (PS3, PM1, PM2, PP3)	Likely Pathogenic (PS3, PM2, PP3)

The five ATP7A variants of unknown significance tested for pathogenicity (patients I to V) are listed in ascending amino acid order. The table also includes the benign control variant (p.E840V) and the three previously reported pathogenic HMNX variants (p.A991D, p.T994I and p.P1386S) for comparison. The final row summarises the ACMG classifications of all variants prior to the incorporation of the functional evidence from this study.

## Results

### Variants causing HMNX cluster within transmembrane domains of ATP7A and exhibit variable effects on protein expression

ATP7A transporters belong to the P_IB_ subclass of heavy metal pumps of the P-type ATPase superfamily. The structure of P_IB_-ATPases is characterized by eight transmembrane spanning helices (MA, MB and M1 to M6) and six heavy-metal binding domains (HMBD1-HMBD6) located at the N-terminus. The catalytic cycle is driven by autophosphorylation and dephosphorylation events catalysed by the actuator (A), phosphorylation (P) and nucleotide binding (N) domains [19]. HMBD6 and these key functional domains of ATP7A harbour a high proportion of the variants reported in MNK patients [20, 21]. By contrast, *ATP7A* variants associated with HMNX cluster on the luminal side of the trans membrane helices M4 (p.A991D and p.T994I) and M6 (p.P1386S), without directly affecting the core catalytic domains of the transporter (Supplementary Fig. 1). The close proximity of these residues in the ATP7A 3D structure, suggests that this region may be critical for the proper function of ATP7A in motor neurons.

**Figure 1:**
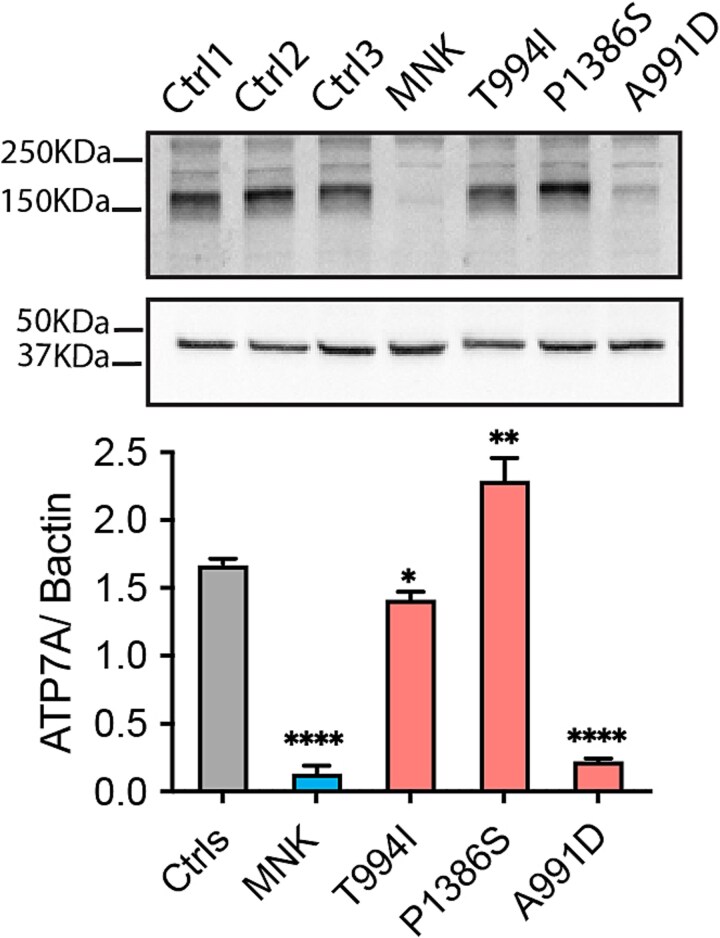
Analysis of ATP7A protein abundance of known HMNX associated variants. Western blot analysis of ATP7A protein levels in fibroblast lysates from HMNX patients and controls. β-actin was used as a loading control. Quantification of ATP7A expression (lower panel) is presented as the mean ± SEM. Data from three control lines were grouped and values were averaged and shown in the control column. Mean ± SEM values are shown for HMNX pathogenic VUS variants and MNK (p.F659Lfs^*^23). Statistical significance was determined by two-way ANOVA followed by Tukey’s multiple comparations test. ^*^P < 0.05; ^**^P < 0.01; ^****^P < 0.0001.

Nearly 25% of MNK-associated variants abolish protein synthesis, by disrupting *ATP7A* splicing [22]. Previous work showed the p.T994I variant causes a mild reduction in ATP7A protein expression in HMNX cell models [13, 14]. To determine whether altered protein abundance is a shared feature in HMNX, human dermal fibroblasts (HDFs) from individuals carrying the three known HMNX variants and from three age- and gender-matched neurologically normal controls were cultured and ATP7A protein levels assessed by western blot analysis (Fig. 1 and Supplementary Fig. 2). Quantification revealed subtle but statistically significant reduction of ATP7A in the p.T994I fibroblasts. The p.A991D missense variant showed a more marked reduction in ATP7A expression, while the p.P1386S variant led to increased levels of ATP7A in fibroblasts. These findings suggest that HMNX causative variants do not consistently reduce ATP7A protein levels, and that simple protein quantification in HMNX-derived fibroblasts is not a reliable stand-alone assay for assessing ATP7A variant pathogenicity.

### Development of a functional assay that defines a common HMNX molecular signature across ATP7A variants

Previous studies with MNK patient fibroblasts have suggested a correlation between intracellular trafficking and severity of the disease [22] with most MNK variants exhibiting impaired Cu-induced trafficking of ATP7A from the TGN. In our previous work on the p.T994I HMNX variant, ATP7A trafficking from the TGN compartment in response to Cu treatment was preserved in patient fibroblasts [13]. However, we observed a significant reduction of ATP7A within the TGN under Cu depleted conditions using the Cu chelating agent bathocuproinedisulfonic acid (BCS). To systematically assess ATP7A intracellular localization of the HMNX variants under different Cu conditions, an automated pipeline was devised using ATP7A immunostaining and confocal imaging directly in 96-well plates, followed by automated image analysis. The experimental design included testing three Cu-loading conditions: Cu-depletion (200 μM BCS), low Cu (FDMEM, containing background levels of trace elements [23], roughly 1.5 μM Cu^2+^) and high Cu (200 μM CuCl_2_). Following treatment, cells were fixed, immunostained for ATP7A and the trans-Golgi TGN marker, golgi-97, and imaged by confocal microscopy. ATP7A localisation to the TGN was quantified in 300–500 cells per condition using automated segmentation of the Golgi region with CellProfiler 4.0.6 as detailed in the *Material and Methods*. Using p.T994I fibroblasts as a positive control, we observed a mild reduction in ATP7A localisation at the TGN under low copper conditions, which became statistically significant following treatment with BCS (Supplementary Fig. 3). Under this non-physiological experimental condition of Cu restriction, our automated assay sensitively and reproducibly detects the characteristic reduction of ATP7A at the TGN.

**Figure 2:**
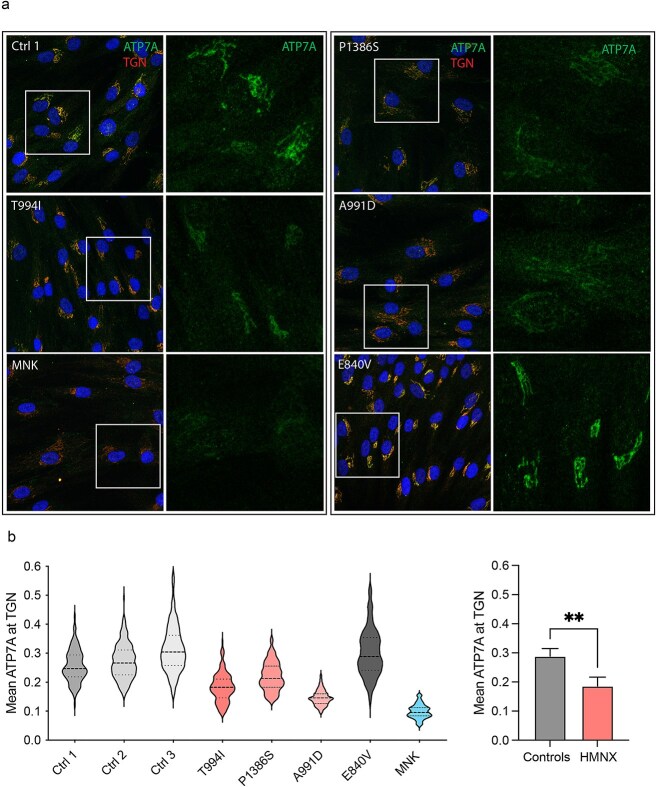
ATP7A levels at the trans-Golgi network (TGN) in HMNX patient fibroblasts under Cu chelating conditions. (a) Representative immunofluorescence images for control, HMNX and MNK fibroblasts stained for ATP7A, TGN marker (golgin 97) and nuclei (DAPI). Insets show a magnified view of the ATP7A signal only to facilitate visualization of the localization pattern across multiple cells; the TGN channel is omitted in the inset images for clarity. (b) Violin plot (left panel) display the distribution of mean ATP7A signal intensity at the TGN across n > 500 ROIs per cell line following 2 h exposure to 200 μM BCS for a representative experiment. The bar graph (right panel) represents as mean ± S.E.M. for all control (grey) and HMNX (red) lines combined. Statistical analysis was performed using a two-tailed Student’s *t-*test (^**^P < 0.01).

Using these optimised conditions, experiments were repeated with the same causative HMNX variants and neurologically normal controls, however a non-pathogenic *ATP7A* variant (p.E840V) and skin fibroblasts from a MNK patient were included as additional controls (Fig. 2). Automated imaging and quantification (>500 cells per variant) following Cu chelation confirmed previously reported ATP7A HMNX associated variants (p.A991D, p.T994I and p.P1386S) share a common phenotype of reduced ATP7A signal at the Golgi compartment. In contrast, cells harboring the p.E840V variant retained the high ATP7A Golgi staining comparable to controls, whereas the MNK fibroblasts showed profound loss of ATP7A expression under the same conditions. Importantly, quantitative analysis of Golgi-97-defined TGN morphology revealed no intrinsic differences between control and HMNX fibroblast lines, indicating that pathogenic *ATP7A* variants do not alter Golgi architecture. These findings support that reduced ATP7A signal at the TGN reflects altered ATP7A protein localisation rather than a segmentation bias arising from differences in TGN organisation (Supplementary Fig. 4).

**Figure 3:**
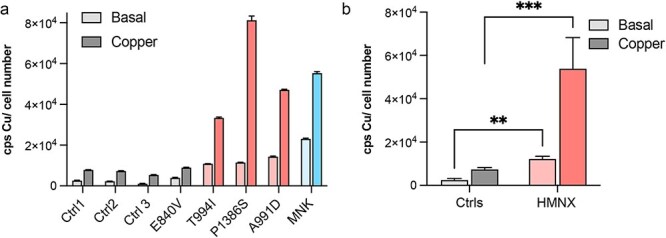
Analysis of intracellular copper in HMNX patient fibroblasts. (a) Intracellular Cu content (cps Cu/cell number) was measured by ICP-MS in control, HMNX and MNK fibroblasts under basal conditions (FDMEM, light columns) or following exposure to 200 μM CuCl_2_, for 16 h (dark columns). Data are shown as counts per second (cps) normalized to cell number and represent the mean ± S.E.M of each technical triplicates. (b) The bar graph represents as mean ± S.E.M. for all control (grey) and HMNX (red) lines combined. Statistical significance was assessed using two-way ANOVA followed by Tukey’s multiple comparisons test. ^**^P < 0.01; ^***^P < 0.001.

Given the essential role of ATP7A in regulating intracellular levels of Cu, we quantified intracellular Cu content in control and patient fibroblasts using inductively coupled plasma mass spectrometry (ICP-MS). All patient derived HMNX cells and MNK fibroblasts showed significant intracellular Cu increases which was exacerbated following 200 μM CuCl_2_ treatment (Fig. 3).

Collectively, our functional characterisation of patient-derived skin fibroblasts demonstrates that quantifying ATP7A levels at the TGN under Cu restricted conditions, together with measuring intracellular Cu accumulation, provides a robust framework for assessing pathogenicity of *ATP7A* variants in HMNX. Furthermore, the clustering of disease-associated residues on the luminal face of transmembrane helices highlights the functional importance of this region for ATP7A-mediated copper transport.

### Functional characterisation of ATP7A variants identifies a novel variant in a HMNX patient

Using our validated functional assay, we next characterised five *ATP7A* VUS (p.R703H, p.Y760C, p.A768G, p.Q990P and p.M1311V) identified in patients (I—V) presenting with distal motor neuropathy and additional neurological features.

**Figure 4:**
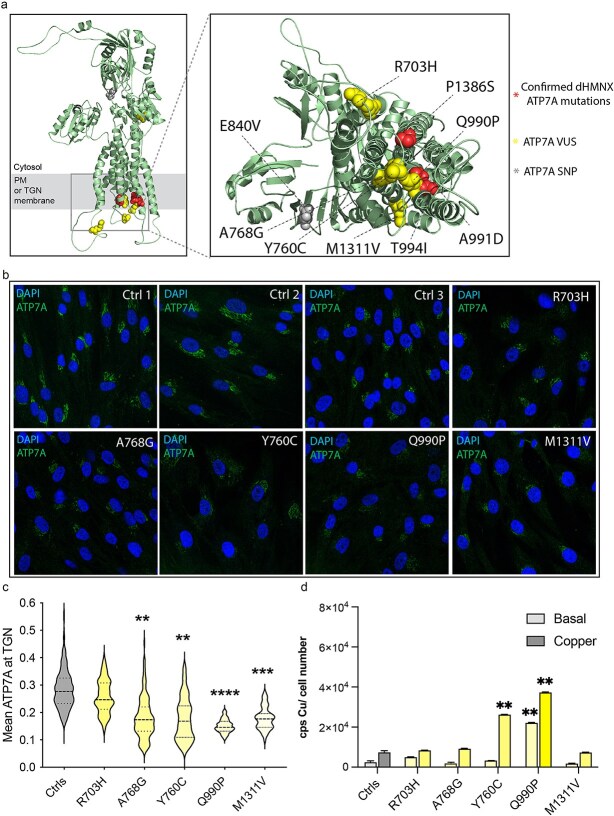
Functional characterisation of ATP7A variants. (a) Homology model of ATP7A showing the positions of previously reported HMNX-associated variants, ATP7A variants and the E840V benign variant in the 3D structure of the Cu transporter. Boxed area is enlarged and re-oriented (view from the extracellular side) to offer a close view of the ATP7A variants and variants used in this study. (b) Representive immunofluorescence images of control and patient fibroblasts bearing *ATP7A* variants (p.R703H, p.A768G, p.Y760C, p.Q990P and p.M1311V) stained for ATP7A following 2 h, 200 μM BCS exposure. (c) Violin plot shows full distribution of all data points (mean ATP7A at the TGN) acquired for each cell line (n > 500 ROIs.), including control samples (three neurologically normal fibroblast lines) and patient-derived fibroblasts. (d) Intracellular Cu content (csp Cu/cell number) measured by ICP-MS in triplicate from control (three neurologically normal fibroblast lines) and HMNX patient fibroblasts with ATP7A variants following incubation under low Cu (FDMEM, light columns) or high Cu (200 μM CuCl_2_, dark columns) conditions for 16 h. data are presented as mean ± S.E.M. statistical significance was assessed by two-way ANOVA with Tukey’s multiple comparisons test; asterisks denote comparisons relative to the control group.

Using the human ATP7A homology model [24] the VUS examined in this study were mapped onto the 3D structure of the transporter. Strikingly, all amino acid residues, except for the p.M1311V (located in the P-Domain) cluster in close spatial proximity to the previously reported HMNX-associated variants. The Tyr760 and the Gln990 amino acid residues reside within transmembrane regions (M1 and M4, respectively), whereas the Arg703 and Ala768 amino acid residues are positioned in the extracytosolic loops between the transmembrane MA-MB and M1-M2 (Fig. 4a).

**Figure 5:**
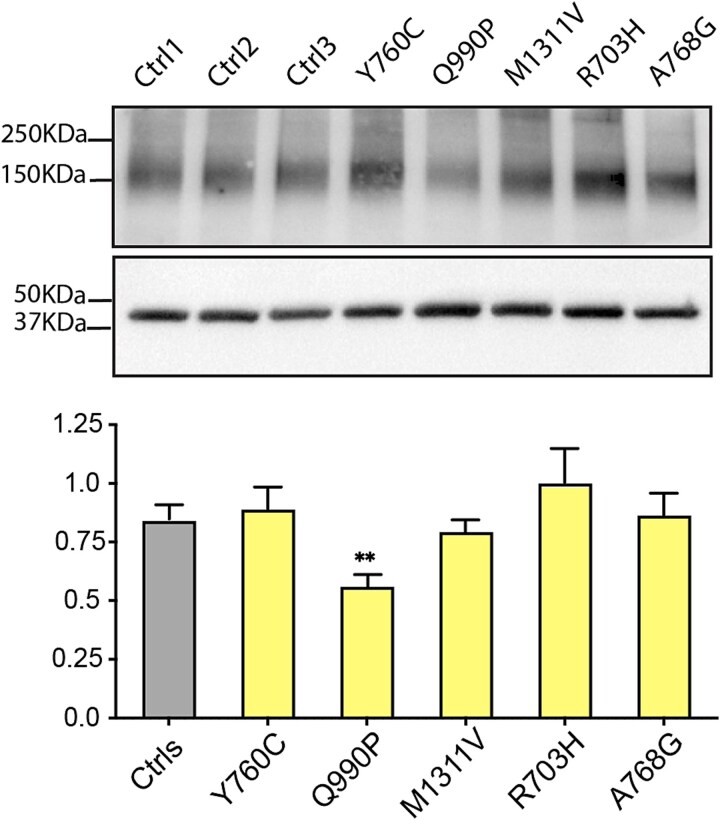
Analysis of ATP7A protein abundance of ATP7A VUS analyised in this study. Western blot analysis of ATP7A protein levels in fibroblast lysates from patients and controls. β-actin was used as a loading control. Quantification of ATP7A expression is shown in the lower panel. Data from three control lines were grouped and values were averaged and shown in the control column (mean ± SEM) folloed by values for *ATP7A* VUS. Statistical significance was determined by two-way ANOVA followed by Tukey’s multiple comparations test. ^**^P < 0.01.

Using our automated imaging pipeline, we quantified ATP7A levels at the TGN in patient-derived fibroblasts (p.R703H, p.A768G, p.Y760C, p.Q990P, p.M1311V) and controls, following chelation of Cu (200 μM BCS). Analysis of acquired images (Fig. 4b) revealed a profound reduction in the levels of ATP7A for the p.Q990P and p.M1311V variants, a mild but statistically significant difference for the p.Y760C and p.A768G missense variants and no detectable changes for the p.R703H variant (Fig. 4c). Intracellular Cu content, measured by ICP-MS under basal (1.5 μM CuCl_2_) and Cu loading (200 μM CuCl_2_) conditions showed that p.Q990P was the only variant associated with significant Cu accumulation in both conditions (Fig. 4), mirroring the phenotype of previously described HMNX-associated variants (Fig. 3). Additionally, analysis of total ATP7A protein levels by Western blot in fibroblast cell lines derived from patients I–V indicated that cells carrying the p.Q990P variant were the only ones showing reduced ATP7A expression/stability (Fig. 5 and Supplementary Fig. 5). Taken together, these results provide strong functional evidence for pathogenicity (PS3 as per the The American College of Medical Genetics and Genomics and Association for Molecular Pathology clinical variant interpretation guidelines, ACMG/AMP) of the p.Q990P (c.2969A > C) genetic variant.

### Clinical, neurophysiological and genetic characterisation of the patient with the ATP7A c.2969A > C (p.Q990P) variant

Patient IV presented with a childhood-onset progressive motor neuropathy initially manifesting as ankle weakness and poor athletic performance at age 11. By age 21, he developed bilateral foot drop and quadriceps atrophy. Over the subsequent decade, his symptoms progressed to include more pronounced bilateral foot drop, distal upper limb weakness, and proximal lower limb weakness with muscle wasting. Neurological examination revealed severe distal weakness in the upper extremities with wrist extension involvement and global lower limb wasting, particularly of the intrinsic foot muscles.

Nerve conduction studies were conducted in the right upper and both lower limbs. Sensory nerve conduction was preserved across all tested sites, and sympathetic skin responses were present bilaterally in the feet, indicating intact sensory and autonomic pathways. In contrast, motor responses were either absent or markedly reduced throughout, consistent with a selective motor involvement. Needle electromyography (EMG) revealed clear evidence of ongoing denervation and reinnervation changes, particularly in the tibialis anterior and medial gastrocnemius muscles. These findings included an absence of normal motor unit recruitment patterns, supporting a chronic motor axonopathy. All neurological and neurophysiological data for patient IV can be found in Supplementary Table 1.

**Figure 6:**
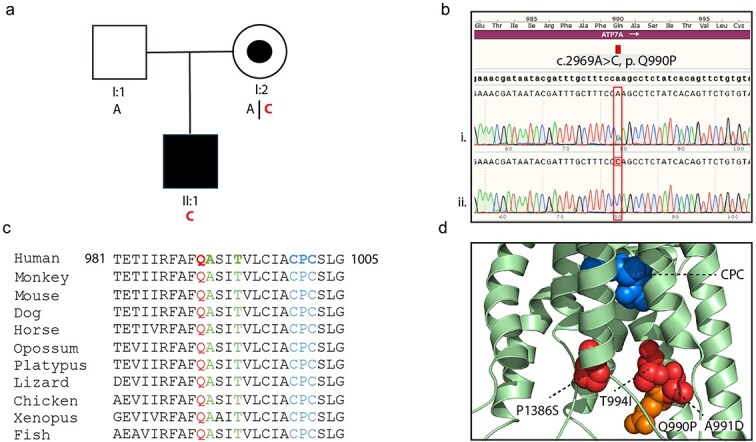
Genetic and functional characterisation of the ATP7A c.2969A > C (p.Q990P) variant. (a) Pedigree of a two-generation family carrying the *ATP7A* c.2969A > C variant. Solid square denotes the affected male (II:1), an open square denotes an unaffected male (I:1), and a circle with a solid central dot denotes an unaffected carrier female (I:2). Genotypes are shown below individuals, with the HMNX-associated variant bolded. (b) Sanger sequencing chromatograms showing the hemizygous and heterozygous *ATP7A* c.2969A > C variant in the affected proband (II:1, panel ii) and the unaffected carrier mother (I:1, panel i). Genbank sequence NM_000052.7 was used as the reference for the *ATP7A* coding sequence. The variant is boxed. (c) Multiple sequence alignment of ATP7A orthologs from human to fish, illustrating strong conservation of the region surrounding residue Q990 (bolded). The amino acid residues affected by two previously described HMNX associated variants in this region of TM6 (p.A991D and p.T994I) and the highly conserved CPC motif (residues 1000 – 1002), essential for Cu co-ordination, have also been bolded. (d) Structural homology model of ATP7A illustrating the spatial clustering the HMNX associated residues. Previously reported pathogenic variants (p.A991D, p.T994I, p.P1386S), the newly identified p.Q990P variant, and the CPC motif are depicted as spheres, highlighting their close proximity within the transmembrane region critical for Cu transport.

Whole exome sequencing of an index patient using a peripheral neuropathy gene panel identified a hemizygous missense mutation (c.2969A > C; p.Q990P) in exon 15 of the *ATP7A* gene. Sanger sequencing confirmed the unaffected mother was heterozygous for the mutation (Fig. 6a and b). *In silico* analysis supported pathogenicity of the variant (Table 1). The missense change identified lies in the same ATP7A a-helix/TM domain (TM6) as the reported c.2981C > T [9] (p.T994I) and c.2972C > A [10] (p.A991D) pathogenic variants and involves a highly conserved proline residue (Fig. 6c). The amino acid sequence surrounding the Pro990 residue is highly conserved across species from human (*Homo Sapiens*) to fish (*Stickleback*) and includes the evolutionary conserved CPC (Cys-Pro-Cys) motif. This assists in Cu binding that is required for Cu transport [25] and is hence critical for ATP7A activity [21, 26–28]. The proximity of the additional pathogenic variant (c.2981C > T; p.P1386S [9]) in the 3D structure of ATP7A may suggest a *structural hotspot* for HMNX pathogenic variants in this region of the Cu transporter (Fig. 6d).

## Discussion

We performed functional assessment of three confirmed *ATP7A* variants (p.A991D, p.T994I and p.P1386S) in patient-derived HMNX fibroblasts. Although HMNX primarily affects motor neurons, ATP7A-mediated copper transport is a fundamental cell-autonomous process conserved across cell types. Patient-derived fibroblasts have been widely used as a clinically accessible model to assess ATP7A function in disorders of copper metabolism, including Menkes disease [22] and ATP7A-related neuropathies [9]. This system enables evaluation of ATP7A trafficking and copper handling in a genetically native context.

**Figure 7:**
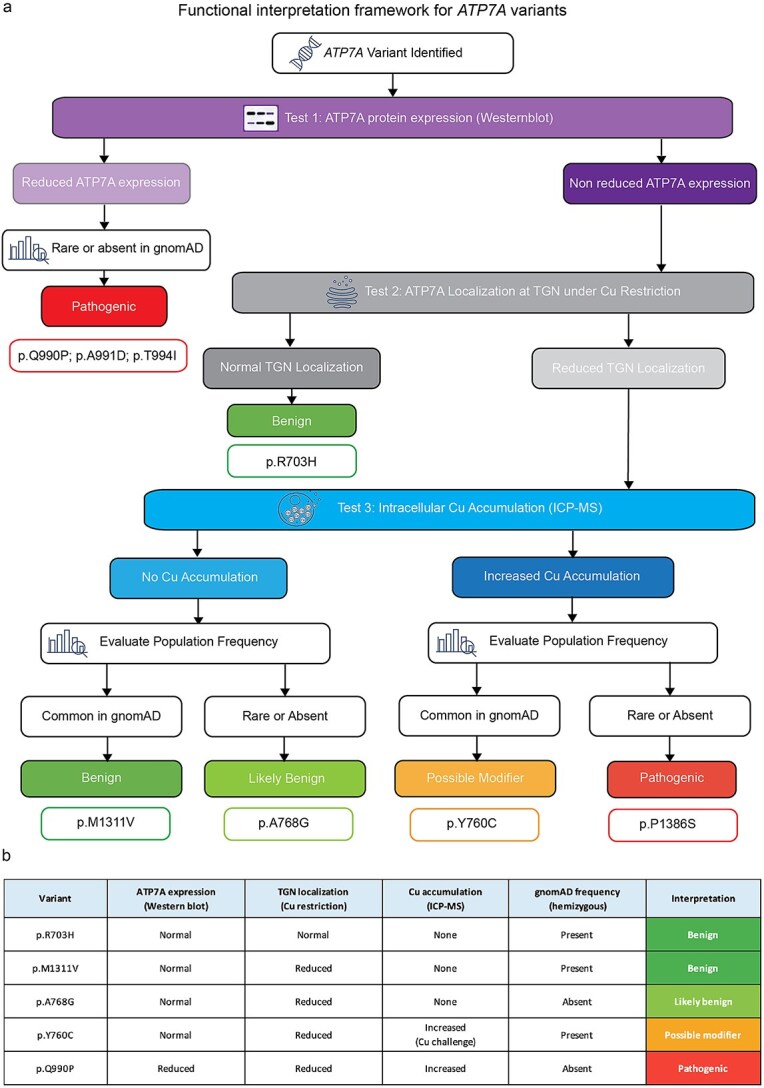
Functional interpretation framework for ATP7A variants. (a) *ATP7A* variants are evaluated using three complementary functional assays: ATP7A protein expression using western blot analysis, ATP7A localization at the trans-Golgi network (TGN) under copper-restricted conditions and intracellular copper accumulation quantified by ICP-MS. these functional readouts are considered alongside population frequency data from gnomAD. Integration of these data enabled classification of variants as benign (p.R730H; p.M1311V), likely benign (p.A768G), possible modifiers (p.Y760C), or pathogenic (p.Q990P). (b) Summary of functional assay outcomes, gnomAD allele frequency and the resulting integrated interpretation for each *ATP7A* variant analysed in this study.

In this work we developed an integrated framework to guide the functional interpretation of *ATP7A* variants (Fig. 7a). This approach combines three complementary cellular readouts (total ATP7A protein levels, ATP7A localisation at the TGN under Cu-restricted conditions and intracellular Cu accumulation following copper challenge) with population frequency data. Our results support a model in which ATP7A protein abundance serves as a highly informative initial indicator of pathogenicity when reduced. Within this framework, impaired ATP7A localisation serves as a sensitive indicator of disrupted trafficking, while intracellular Cu accumulation reflects reduced Cu export capacity. Integration of these functional assays with gnomAD allele frequency allows stratification of *ATP7A* variants into benign, likely benign, possible modifiers, or pathogenic categories (Fig. 7b). Importantly, this framework also provides a rationale for interpreting discordant results between assays, highlighting that partial functional defects may not be sufficient to confer pathogenicity in the absence of supporting genetic evidence. Together, this model refines the interpretation of *ATP7A* variants in a clinically relevant context.

Applying our integrated framework to five *ATP7A* VUS, we generated functional evidence that enabled classification of novel variant, p.Q990P, as disease causative in a patient with progressive peripheral motor neuropathy. This work further expands the genetic heterogeneity of ATP7A-related neuropathies.

Using a homology model of human ATP7A, we showed all four confirmed HMNX associated variants cluster in the transmembrane region of the Cu transporter in helices M4 and M6 near the luminal side. Although these residues likely line the release pathway used for Cu transport across the membrane, the functional effect is clearly milder than mutations of the CPC-motif [27]. The shared localisation and functional defects of the HMNX residues underscore the importance of this region for ATP7A activity in motor neurons. Pathogenic mechanisms proposed for HMNX associated variants have been linked to ATP7A intracellular trafficking [29] defects, either through disruption of interactions with adaptor protein complexes AP1 and AP2 (as described for p.P1386S) [12] or through a toxic gain of interaction with VCP/97 for p.T994I [30]. Previous work suggests the p.T994I substitution induces conformational change that exposes a previously hidden conserved hydrophobic Phe-Pro bipeptide (residues 971–972) within the UBX domain of ATP7A, thereby enhancing ATP7A-p97/VCP binding [30]. A similar mechanism may underlie the pathogenicity of p.Q990P and p.A991D. Structurally, p.Q990P introduces a proline residue within the transmembrane region of ATP7A, which may disturb local protein conformation and stability, and simultaneously create a new Phe-Pro motif at positions 989–990, that could augment the existing ATP7A UBX domain (positions 971–972) which interacts with the ubiquitin-selective chaperone p97/VCP. Additional functional evidence would be needed to test this hypothesis directly.

An increasing number of *ATP7A* variants are being identified in motor neuropathy patients and reported in the literature. Notably, these individuals often present with additional and more complex clinical features, including type III spinal muscular atrophy (SMA) [15], Wilson disease [16], and OHS [17]. This clinical heterogeneity together with the paucity of functional studies underscores the need for robust assays to assess the pathogenicity of *ATP7A* variants. In this study we applied our functional pipeline to fibroblasts from patients with distal motor neuropathy and other more complex neurological manifestations. In addition to distal motor neuropathy, individual V showed tongue fasciculations, Babinski signs and cerebellar ataxia.

The p.M1311V (c.3931A > G) variant identified in this patient was recently reported in a male of Jewish ancestry [31] with amyotrophic lateral sclerosis (ALS) where defects in ATP7A intracellular trafficking were described in patient derived fibroblasts without affecting ATP7A protein expression [32] (Fig. 5 and Supplementary Fig. 5). M1311 is positioned adjacent to a region suggested to play a modulatory role in P_IB_-ATPases [33], making it difficult to completely exclude the possibility that the p.M1311V missense variant influences ATP7A function. However, our functional assays do not support a pathogenic role for p.M1311V in the context of HMNX (Fig. 4) as intracellular Cu levels were maintained across different Cu loading conditions. Consistent with this, the M1311 residue lies within the P-Domain of ATP7A, spatially distinct from the cluster of confirmed HMNX-associated variants. Population data from gnomAD v3 report a hemizygote frequency of 2.16% for p.M1311V in the Ashkenazi Jewish males (17/786 alleles) which is markedly higher than the reported prevalence of ALS (4.1 and 8.4 per 100 000 persons) [34] or HMN (2.1 per 100 000 individuals) [35]. Yeomin Yun et al. concluded that additional genetic variants should be investigated in their ALS patient. Our functional data in fibroblasts carrying the same variant supports this view and further calls into question the pathogenic role of p.M1311V in neurodegenerative disease.

Individual II in our study presented with an unusual complex phenotype resembling Wilson disease [16], including axonal motor neuropathy, dystonia and ataxia (see Bansagi, B. *Neurol Genet* 2016 for full phenotypic and clinical information [16]). When the p.Y760C (c.2279A > G) variant was identified in 2016, it was considered rare (Exome Aggregation Consortium: 4 heterozygous X chromosomes among 87 766, no hemizygotes). Investigation of ATP7A protein expression in that study showed a marked reduction of ATP7A in the patient fibroblasts [16]. Assessment of ATP7A expression using our experimental conditions however demonstrates no changes in protein levels in cells obtained from this patient (Fig. 5 and Supplementary Fig. 5). Our functional studies in patient-derived p.Y760C fibroblasts showed a mild but significant reduction of ATP7A levels at the TGN and intracellular Cu accumulation, but only after challenge with 200 μM CuCl_2_ (Fig. 4). Although the Tyr760 residue is distant from the other HMNX-associated variants in the primary sequence, mapping it onto the ATP7A homology model showed this residue, located in the transmembrane domain M1, lies only 5.9 and 7.0 angstroms (Å) from the p.T994I and P.P1386S respectively (Supplementary Fig. 6). Interestingly, the neighboring p.S761P substitution has been associated with a moderate MNK phenotype [8]. Taken together, our functional data and the updated gnomAD v3 frequency for p.Y760C (22/1208816 heterozygous X chromosomes, and 8 hemizygotes) do not strongly support a primary pathogenic role for this variant but raises the possibility that p.Y760C may act as a genetic modifier contributing to the complex phenotype observed in this patient. Increasing evidence indicates that variants with modest functional effects, may not be sufficient to cause disease independently, but can influence phenotypic expression, disease severity, or age of onset in the presence of a primary pathogenic allele. In this context, modifier or risk alleles represent an important component of the genetic architecture of inherited neuropathies, bridging the continuum between classical Mendelian and more complex inheritance models [36]. Our findings align with this paradigm and support a more nuanced interpretation of *ATP7A* variation, whereby partial functional defects may contribute to disease in a context-dependent manner rather than acting as fully penetrant causal mutations.

Our experiments unambiguously exclude the p.R703H and p.A768G variants detected in patients I and III as disease causing. These results align with the work by A. Ogawa et al. which described the p.R703H (c.2108G > A) variant as a polymorphism in the Japanese population [37]. In line with this classification, our experiments showed these variants do not affect ATP7A protein expression or stability in fibroblasts obtained from these patients (Fig. 5 and Supplementary Fig. 5).

Our experiments show that markedly reduced expression of ATP7A is associated with HMNX development (p. A991D, p. T994I and the newly described p. Q990P, Figs 1 and 5), suggesting that assessment of global ATP7A protein expression by Western blot analysis, remains as a valuable initial tool to inform pathogenicity of *ATP7A* variants. Importantly, our results demonstrate that normal or even elevated protein levels (p.P1386S, Fig. 1) do not exclude pathogenicity and therefore this assay cannot be used as a reliable discriminator between benign and pathogenic variants. Our study provides a functional framework for assessing novel *ATP7A* variants in these cases. We also show that HMNX-causing *ATP7A* variants cluster within a common structural region of the Cu transporter and share a characteristic molecular signature, which together may provide a tool for variant classification in a diagnostic setting. While our data provide strong evidence that p.Q990P is a pathogenic variant and further defines the molecular phenotype of *ATP7A* variants associated with HMNX, several questions remain. Firstly, additional studies in neuronal models or patient-derived motor neurons are needed to clarify how ATP7A dysfunction contributes to axonal degeneration in HMNX. Secondly, our work focused on ATP7A localization and intracellular copper levels. Further studies should investigate ATP7A protein–protein interactions, to directly test whether p.Q990P and p.A991D alter binding to p97/VCP.

## Materials and methods

### Research guidelines and regulations

All research and cell culture procedures were conducted following written consent according to protocols approved by the Sydney Local Health District Human Ethics Review Committee, Concord Repatriation General Hospital, Sydney, Australia (reference number: HREC/11/CRGH/105). Informed consent for study participation was obtained from all patients and controls. All research was performed in accordance with relevant guidelines and regulations.

### Human primary fibroblasts cultures

Human dermal fibroblasts (HDFs) were cultured from skin biopsies of three clinically normal subjects, HMNX patients harboring the p.T994I, p.P1386S, and p.A991D ATP7A variants and VUS (p.R703H, p.Y760C, p.A768G, p.Q990P and p.M1311V) in individuals diagnosed with distal motor neuropathy. HDFs with a non-pathogenic *ATP7A* SNP (E840V) and a MNK patient (p.F659Lfs^*^23) were included as additional controls. HDFs were cultured in F-DMEM media consisting of DMEM media (Gibco, Life Technologies) supplemented with 10% (v/v) fetal bovine serum (SAFC Biosciences), 1% (v/v) Penicillin Streptomycin and 1% (v/v) L-glutamine (Gibco, Life technologies) at 37°C under humidified air with 5% CO_2_.

### Western blotting

Cell lysates were obtained from HDFs (1 × 10^6^ cells) using RIPA buffer (50 mM Tris-HCl pH 8.0, 150 mM NaCl, 0.1% w/v SDS, 1% v/v Triton X-100, 1% w/v Sodium deoxycholate, 1× cOmplete, Mini EDTA-free protease inhibitor). After protein quantitation (Pierce BCA Protein Assay Kit, ThermoScientific) cell lysates (20 μg) were subjected to SDS-polyacrylamide gel electrophoresis and transferred to polyvinylidene difluoride (PVDF) membranes. Membranes were probed with a rabbit polyclonal ATP7A antibody (Auspep) raised specifically against a peptide corresponding to amino acids 1463–1480 at the carboxy-terminus of human ATP7A (DKHSLLVGDFREDDDTAL). Alpha tubulin (SIGMA Aldrich; T5168; 1:5000) and β-actin (Cell Signaling; #4967; 1:2000) antibodies were used as loading controls. Anti-rabbit (SIGMA Aldrich; A0545; 1:10000) and anti-mouse (Abcam; ab97023; 1:5000) horseradish peroxidase (HRP) conjugated secondary antibodies were used and signal detection was performed using a chemiluminescent substrate (Merck). Densitometric quantification of western blot images was performed from three independent experiments using the open-source platform for biological-image analysis Fiji [38]. Briefly, a region of interest (ROI) was drawn around the target protein band and an intensity plot generated. The same ROI selection frame was used to define protein bands across all samples. The line selection tool was used to delineate the boundaries of the intensity peaks corresponding to protein bands, and the area of each peak was then measured. GraphPad Prism (version 10) was used to generate graphs and calculate statistical significance, using a two-way ANOVA followed by Tukey’s multiple comparations test.

### Immunofluorescence

HDFs (1.5 × 10^4^) for each patient and control were plated in optically clear bottom 96-well plates (CellCarrier-96, PerkinElmer). After 48 h, HDFs were exposed to Cu (200 μM CuCl_2_) or Cu chelation (200 μM bathocuproinedisulfonic acid, BCS) for 2 h at 37°C. HDFs were then washed once using PBS, fixed with 4% (v/v) paraformaldehyde (PFA) for 12 min at room temperature (RT), permeabilized in phosphate-buffered saline (PBS) containing 0.3% (v/v) Triton X-100 and blocked in 5% (w/v) bovine serum albumin (BSA) for 60 min. Cells were incubated with the primary antibodies overnight at 4°C (rabbit monoclonal ATP7A 1:250, Antibody Solutions; mouse monoclonal golgin 97 CDF4 1:500, Santa Cruz) and with Alexa Fluor-labeled secondary antibodies (Molecular Probes-Invitrogen, Paisley, UK) at RT for 2 h. Nuclei were counterstained with 300 nM 4,6-diamidino-2- phenylindole (DAPI, Molecular Probes).

### Quantification of ATP7A present at the TGN

Cells were visualized using a Leica SP8 confocal microscope equipped with a motorised stage for automated acquisition of images. Images were acquired at 20× magnification and 3× digital zoom. For quantification of the ATP7A present at the TGN following treatment, settings for optimal visualization of ATP7A at the TGN in control samples and Cu-chelating conditions were determined and used for the acquisition of all subsequent samples in the experiment. Thirty or more images were acquired for each condition (300–500 cells). To precisely define the region of interest in each image corresponding to the TGN and specifically quantify the presence of ATP7A at that location, segmentation of the trans-Golgi network for each cell was achieved by using the open-source software image analysis, CellProfiler 4.0.6 (https://cellprofiler.org/) and in-house pipelines. Briefly, nuclei and TGN regions were segmented as primary objects using Global (Otsu/two-class) thresholding. Nuclei were masked from the resulting images by applying the *MaskObject* module and the region per cell corresponding to the TGN refined by using the *SplitOrMergeObjects* module (Distance merging method). ATP7A fluorescence intensity was then measured specifically within these segmented TGN objects using the *MeasureObjectIntensity* module. Batch processing was performed across all images to maintain consistent parameter settings. Extracted measurements were exported as CSV files and used for subsequent statistical analysis. Data was plotted using GraphPad Prism version 10.2.3 for visualization and statistical analysis.

### Intracellular metal analysis

Total intracellular metal content was determined in control and patient HDFs after the different treatments using inductively coupled plasma mass spectrometry. Cells (2 × 10^5^) were plated in 100 mm plates and grown to 90% of confluency. On day 5, HDFs were cultured in F-DMEM containing 200 μM CuCl_2_ or non-supplemented F-DMEM for 16 h. The cells were harvested with trypsin (0.1% v/v) pelleted, and washed in PBS (1 mL) twice. An aliquot of resuspended cells (100 μl) was set aside for cell counting (Countess II FL Automated Cell Counter, Life Technologies) to normalise CP-MS data. The remaining cell suspension (0.9 ml) was centrifuged to produce a cell pellet which was analysed for Cu content using ICP-MS. The cell pellets were digested in 0.5 ml high grade HNO_3_ (69%) for 2 h using a water bath set at 65°C. The digest was diluted to 10 ml with HCl (0.1 M). ICP-MS was run on a Perkin Elmer Nexion 2000 Inductively Coupled Plasma Mass Spectrometer (ICP-MS) at the Mass Spectrometry Facility (University of Sydney). Intracellular Cu content is reported in counts per second (cps) Cu atoms/cell.

### Structural data

For 3D visualization of amino acid residues of interest, a homology model for ATP7A [24] was used (based on the structure of the homologous LpCopA from the bacterium *Legionella pneumophila* [21]). Structural images were rendered using PyMOL (htpp://www.pymol.org).

### *In silico* prediction tools

Pathogenic prediction of *ATP7A* variants in this study was perfromed using a series of *in silico* tools including SIGMA [39], REVEL [40], AlphaMissense [41], GERP [42], EVE [43] and SIFT [44]. Minor allele frequency of variants was assessed using gnomAD v3 [45].

### Statistical analysis

Statistical analyses were performed, using data from three independent experiments conducted under the same conditions. Student’s *t test* or 2way ANOVA test with Tukey’s multiple comparations was used to assess statistical significance. Data are presented as mean ± SEM. The following statistical thresholds were applied: ^*^P < 0.05; ^**^P < 0.01; ^***^P < 0.001; ^****^P < 0.0001.

## Supplementary Material

Supplementary_materials_ddag061
