# Supplementary material for: A functional framework in patient fibroblasts informs ATP7A variant pathogenicity and identifies p.Q990P as a novel cause of distal motor neuropathy
Source: Hum Mol Genet. 2026 Jul 8;35(14):ddag061. doi: 10.1093/hmg/ddag061 (PMC13345369; doi:10.1093/hmg/ddag061)
Supplement: Supplementary_materials_ddag061 [file supplementary_materials_ddag061.zip › HMG-25-01256.R1_PerezSiles_SupplementaryTable 1.docx]

# **Supplementary Table 1.**

# Nerve Conduction Study (NCS) Results

## Sensory NCS

| Nerve / Sites | Rec. Site | Amplitude (μV) | Latency (ms) | Distance (cm) | Velocity (m/s) |
| --- | --- | --- | --- | --- | --- |
| R MEDIAN - Ortho Palm | Wrist | 99.5 | 1.6 | 8 | 51.2 |
| R MEDIAN - Ortho Dig II | Wrist | 15.3 | 2.7 | 14 | 51.7 |
| R ULNAR - Ortho Palm | Wrist | 26.0 | 1.4 | 8 | 59.1 |
| R ULNAR - Ortho Dig V | Wrist | 6.7 | 2.1 | 11 | 52.8 |
| R SURAL - Ortho | Calf | 10.3 | 2.0 | 9 | 44.3 |
| L SURAL - Ortho | Calf | 12.3 | 2.0 | 9 | 45.5 |

## Motor NCS

| Nerve / Sites | Amplitude (mV) | Latency (ms) | Dur. (ms) | Area (mVms) | Dist. (cm) | Vel. (m/s) |
| --- | --- | --- | --- | --- | --- | --- |
| R MEDIAN - APB (Wrist) | 1.0 | 3.5 | 7.0 | 3.5 |  |  |
| R MEDIAN - APB (Elbow) | 0.7 | 7.9 | 5.9 | 1.3 | 26 | 58.7 |
| R ULNAR - ADM (Wrist) | 0.4 | 3.5 | 7.0 | 1.8 |  |  |
| R ULNAR - ADM (B. Elbow) | 0.4 | 8.1 | 5.6 | 1.6 | 25.5 | 55.6 |
| R ULNAR - ADM (A. Elbow) | 0.4 | 10.1 | 8.8 | 2.2 | 10 | 50.5 |
| R COMM PERONEAL - EDB | NR | NR | NR | NR | NR | NR |
| L COMM PERONEAL - EDB | NR | NR | NR | NR | NR | NR |
| R TIBIAL MALLEOLUS - FHB | NR | NR | NR | NR | NR | NR |
| L TIBIAL MALLEOLUS - FHB | NR | NR | NR | NR | NR | NR |
| R COMM PERONEAL - Tib Ant | NR | NR | NR | NR | NR | NR |
| L COMM PERONEAL - Tib Ant | NR | NR | NR | NR | NR | NR |
| R ULNAR - FDI | 0.5 | 5.1 | 4.6 | 1.0 |  |  |

# Electromyography (EMG) and Reflex Study Results

## Sympathetic Skin Response (SSR)

| Nerve / Sites | Rec. Site | Latency (ms) | Amplitude (mV) |
| --- | --- | --- | --- |
| L GENERAL NERVE - 1 Channel | Sole | 3625.0 |  |
| R GENERAL NERVE - 1 Channel | Sole | NR | NR |

## F Wave

| Nerve | Min F Lat (ms) | Max F Lat (ms) | % F |
| --- | --- | --- | --- |
| R ULNAR - ADM | Absent |  |  |

## Needle EMG Summary

| Muscle | IA | Fib/PSW | Fasc | H.F. | Amp | Dur. | PPP | Pattern |
| --- | --- | --- | --- | --- | --- | --- | --- | --- |
| R. TIB ANTERIOR | - | 4+ | None | None | No Activity | No Activity |  | No Activity |
| R. GASTROCN (MED) | - | 4+ | None | None | No Activity | No Activity |  | No Activity |
| R. VAST LATERALIS | - | None | None | None | Inc | Inc | 1+ | Single unit |
| R. DELTOID | - | None | None | None | Inc | Inc | 1+ | Reduced |
| R. BICEPS | - | None | None | None | Inc | Inc | 1+ | Reduced |
| R. TRICEPS | - | None | None | None | Inc | Inc | 1+ | Reduced |
| R. FIRST D INTEROSS | - | None | None | None |  |  |  | No Activity |

## Multi motor unit potential (MUP) analysis

| Muscle | Sweeps | Amp (µV) | Dur (ms) | Phases | Turns | Area (µVms) | Ratio | Rate (pps) |
| --- | --- | --- | --- | --- | --- | --- | --- | --- |
| R. DELTOID - Mean ALL |  | 2715 | 16.7 | 2.3 | 4.6 | 6423.4 | 2.29 | 10 |
| R. DELTOID - Mean SIMPLE |  | 2715 | 16.7 | 2.3 | 4.6 | 6423.4 | 2.29 | 10 |
| Total MUPs | 13 |  |  |  |  |  |  |  |
| % Polyphasic | 0% |  |  |  |  |  |  |  |
| % Simple | 100% |  |  |  |  |  |  |  |
