# Supplementary material for: A functional framework in patient fibroblasts informs ATP7A variant pathogenicity and identifies p.Q990P as a novel cause of distal motor neuropathy
Source: Hum Mol Genet. 2026 Jul 8;35(14):ddag061. doi: 10.1093/hmg/ddag061 (PMC13345369; doi:10.1093/hmg/ddag061)
Supplement: Supplementary_materials_ddag061 [file supplementary_materials_ddag061.zip › HMG-25-01256.R1_PerezSiles_SupplementaryData_FINAL.docx]

**Supplementary Figure 1.** *Spatial localization in ATP7A 3D structure of known HMNX associated variants.* The boxed area shows a close up of the luminal side of the trans membrane helices of M4 and M6 with amino acid residues (p.A991, p.T994 and p.P1386) shown as red spheres. The grey band represents the plasma or trans-Golgi network membrane, with the indicated cytosolic side.

**Supplementary Figure 2.** *Western blot analysis of ATP7A protein levels in fibroblast lysates from HMNX patients and controls*. β-actin was used as a loading control.

**Supplementary Figure 3.** *Automated imaging and quantification of ATP7A localisation at the trans-Golgi network (TGN) in human dermal fibroblasts.* **A.** Intracellular Cu levels regulate ATP7A subcellular localization. ATP7A constitutively cycles between the TGN and plasma membrane (PM). Under high copper conditions (200 μM CuCl_2_), there is a steady state shift in ATP7A at the PM, whereas copper depletion (200 μM BCS) promotes ATP7A retention at the TGN. ATP7A trafficking in these conditions is regulated by clathrin-mediated endocytosis and Rab-dependent endosomal trafficking. **B.** Quantitative analysis shows a statistically significant reduction of ATP7A at the TGN in fibroblasts harboring the T994I variant compared to control cells (Ctrl 1), under Cu chelating (200 μM BCS) conditions. ATP7A localization to the Golgi was reduced under basal conditions (FDMEM) for all samples, and while the abundance of the transporter at the TGN was minimal following 200 μM CuCl_2_ exposure, no difference was found between control and patient cells under these conditions. Violin plot displays the distribution of ATP7A intensity data points for the acquired regions of interests (ROIs, n>500 cells/sample). Statistical analysis was performed using two-way ANOVA followed by Tukey’s multiple comparations test. ***p<0.001 **C.** Representative immunofluorescent images of control and HMNX (p.T994I) fibroblasts under the three treatment conditions (200 μM BCS, FDMEM and 200 μM CuCl_2_, for 2 h).

**Supplementary Figure 4.** *Quantitative analysis of TGN morphology in control and HMNX fibroblasts.* **(A)** Representative images of control and HMNX fibroblasts stained with DAPI (blue) and Golgi-97 (red). Trans-Golgi network (TGN) objects were segmented and quantified using automated image analysis in CellProfiler. Violin plots show the distribution of mean ATP7A signal intensity at the TGN across >500 ROIs per cell line with bar graphs representing combined mean ± S.E.M. for all control (grey) and HMNX (red) lines. TGN morphology was quantified using size and shape descriptors, including **(B)** area (µm²), **(C)** compactness, **(D)** eccentricity, **(E)** form factor, **(F)** solidity, and **(G)** equivalent diameter (µm). Area and equivalent diameter reflect TGN size, while eccentricity, solidity, compactness, and form factor capture object shape and structural regularity, including features such as elongation, dispersion, and fragmentation.

**Supplementary Figure 5**. *Analysis of ATP7A protein abundance of ATP7A VUS analyised in this study.* Western blot analysis of ATP7A protein levels in fibroblast lysates from patients and controls. β-actin was used as a loading control.

**Supplementary Figure 6**. Homology model of ATP7A showing the position of the p.Y760C variant (yellow spheres) in relation to the p.T994I and p.P1386S HMNX causing variants (red spheres) in the 3D structure of the Cu transporter. Molecular distances (angstroms; Å) between the residues are calculated with Pymol.
