# Supplementary figures and images for: A functional framework in patient fibroblasts informs ATP7A variant pathogenicity and identifies p.Q990P as a novel cause of distal motor neuropathy
Source: Hum Mol Genet. 2026 Jul 8;35(14):ddag061. doi: 10.1093/hmg/ddag061 (PMC13345369; doi:10.1093/hmg/ddag061)

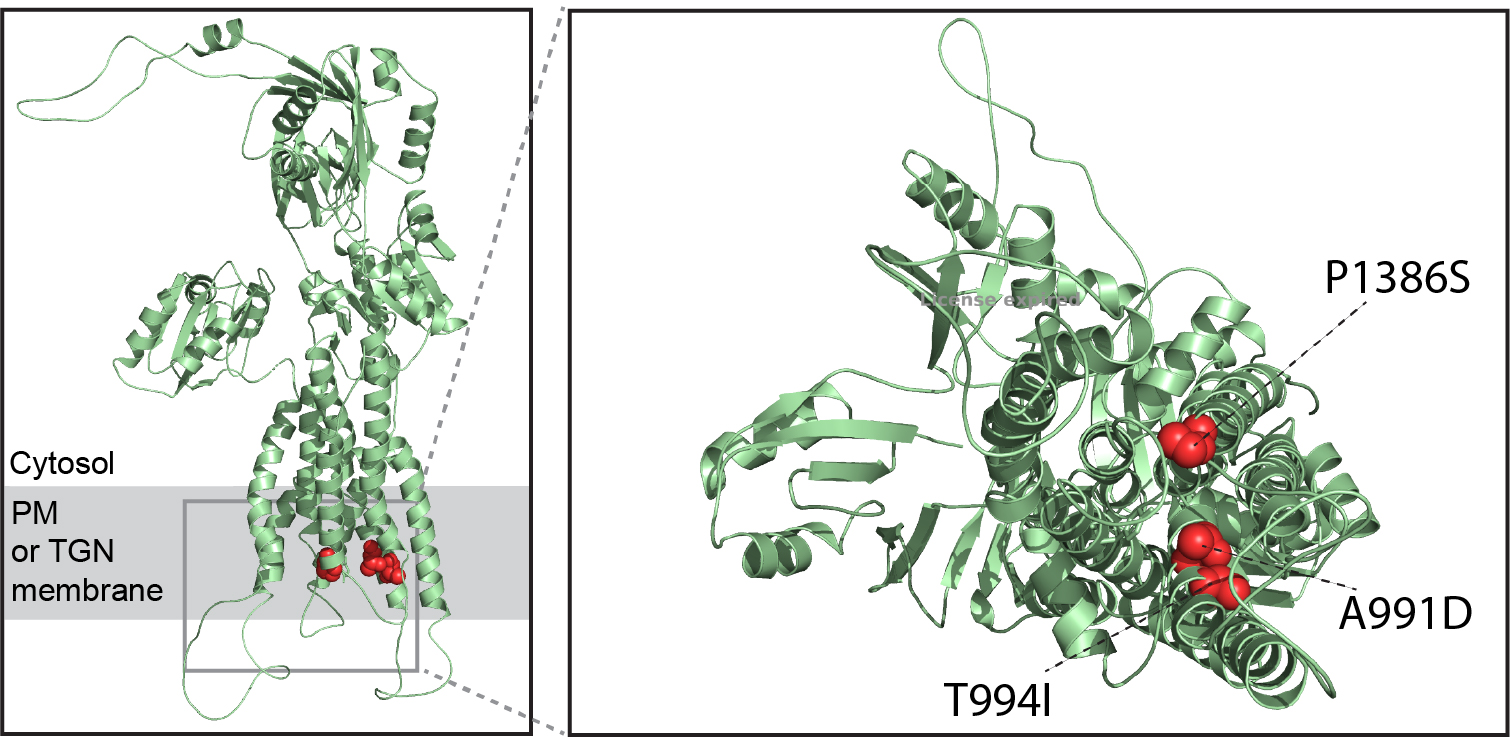

Supplement: Supplementary_materials_ddag061 [file supplementary_materials_ddag061.zip › ATP7A_VUS_SupplemFig1_Revision2.jpg]

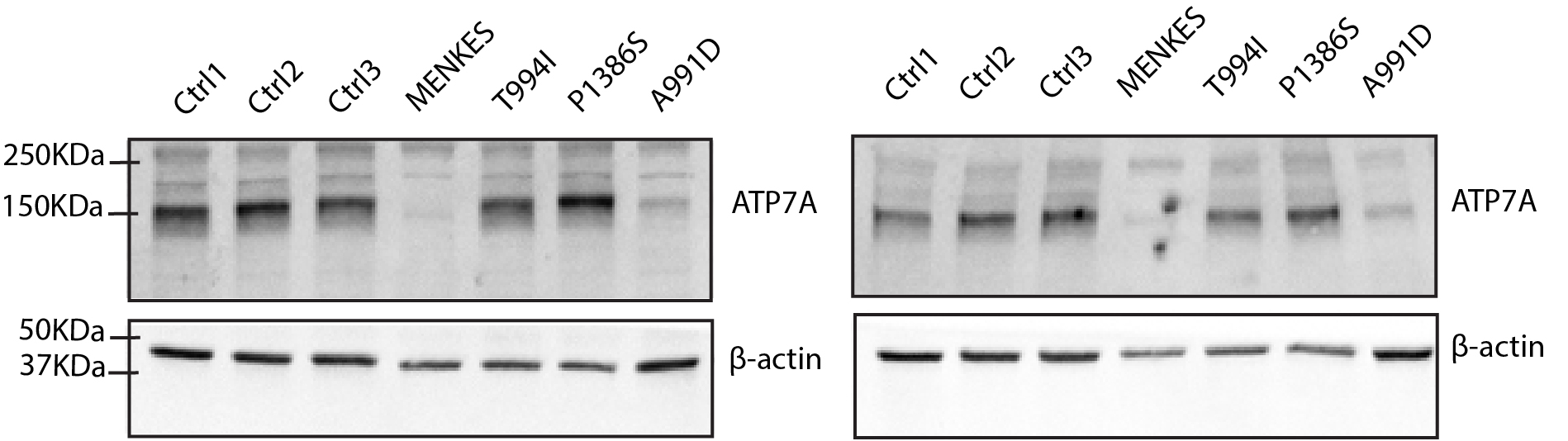

Supplement: Supplementary_materials_ddag061 [file supplementary_materials_ddag061.zip › ATP7A_VUS_SupplemFig2_Revision2.jpg]

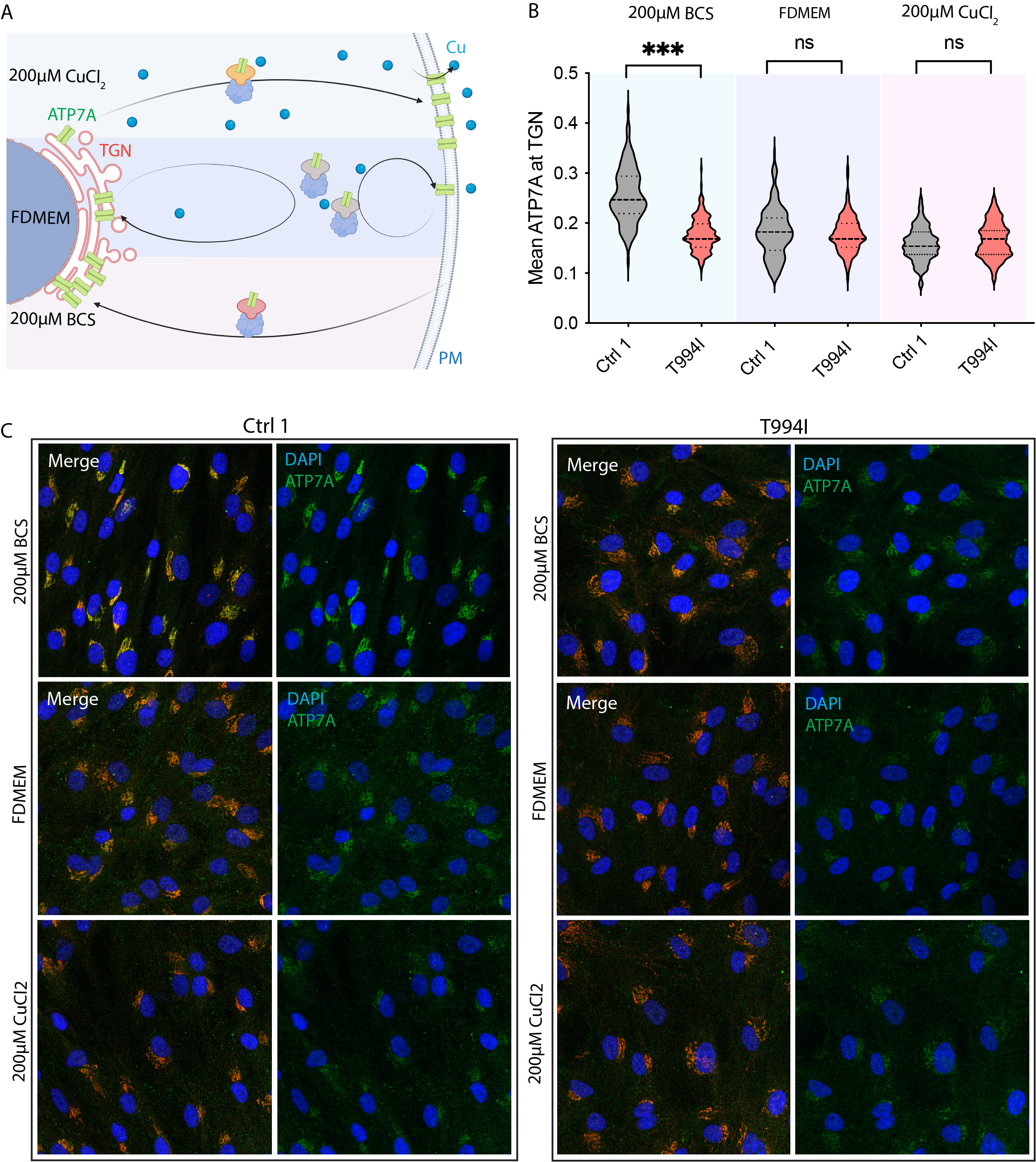

Supplement: Supplementary_materials_ddag061 [file supplementary_materials_ddag061.zip › ATP7A_VUS_SupplemFig3_Revision2.jpg]

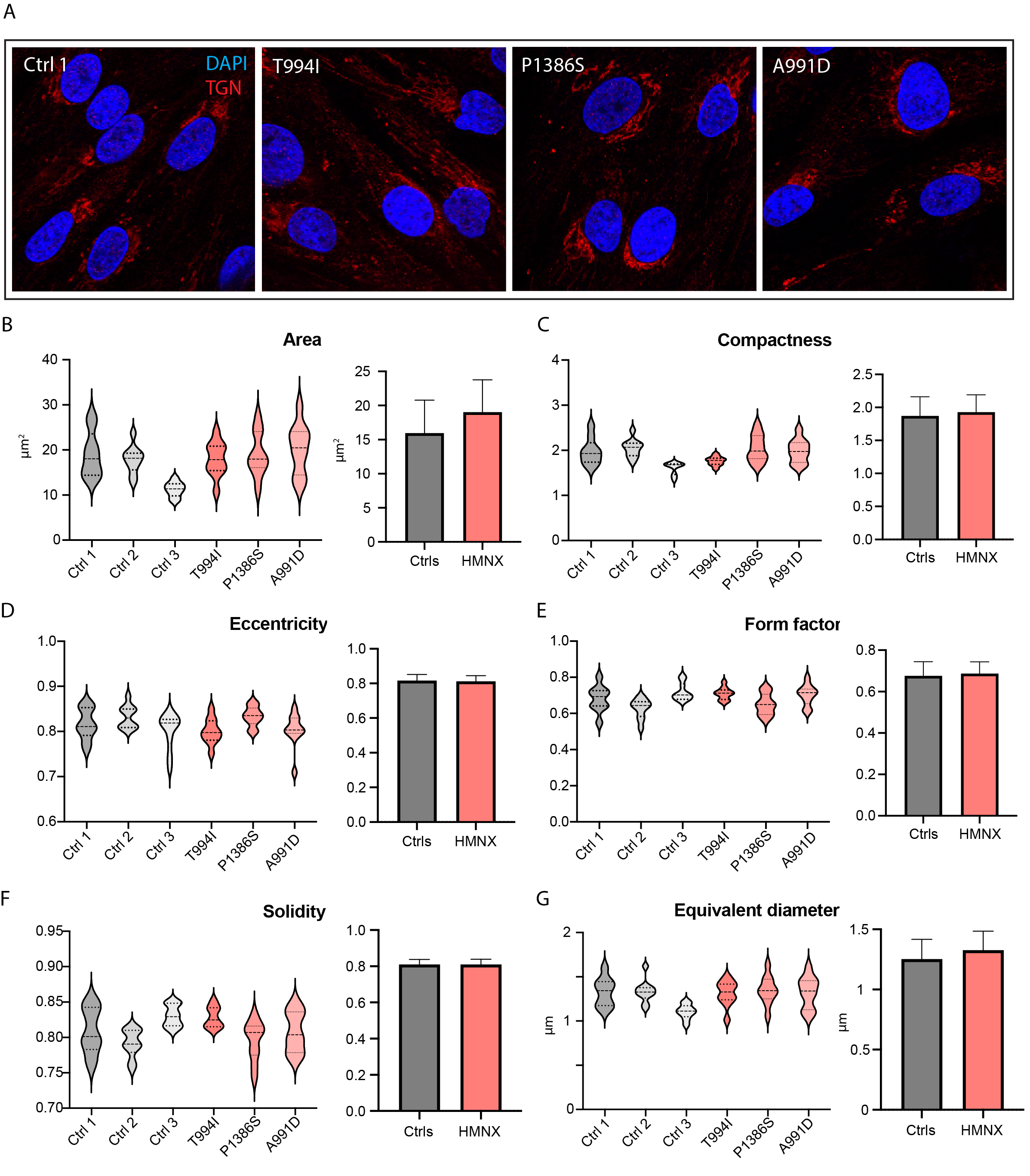

Supplement: Supplementary_materials_ddag061 [file supplementary_materials_ddag061.zip › ATP7A_VUS_SupplemFig4_Revision2.jpg]

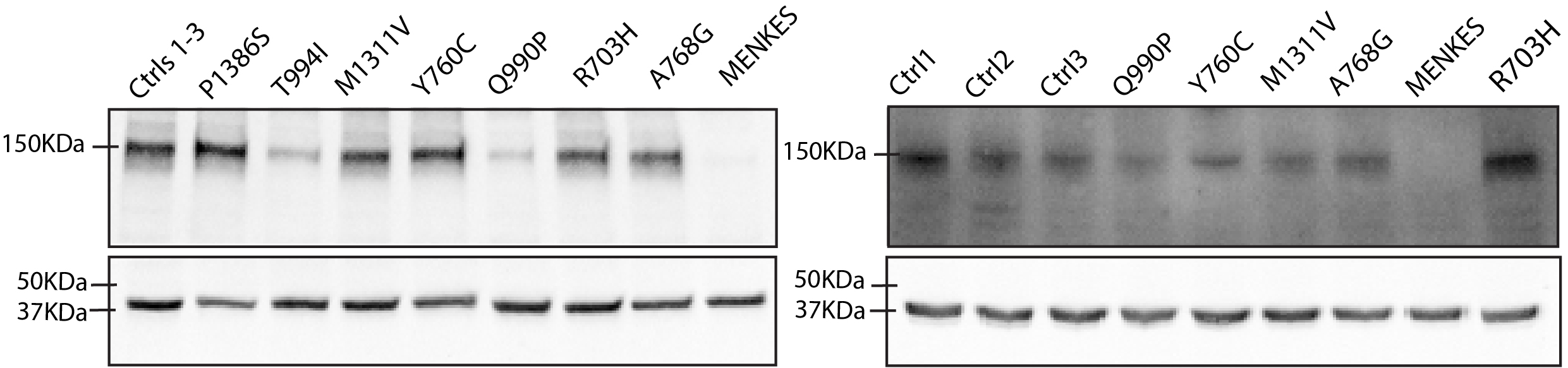

Supplement: Supplementary_materials_ddag061 [file supplementary_materials_ddag061.zip › ATP7A_VUS_SupplemFig5_Revision2.jpg]

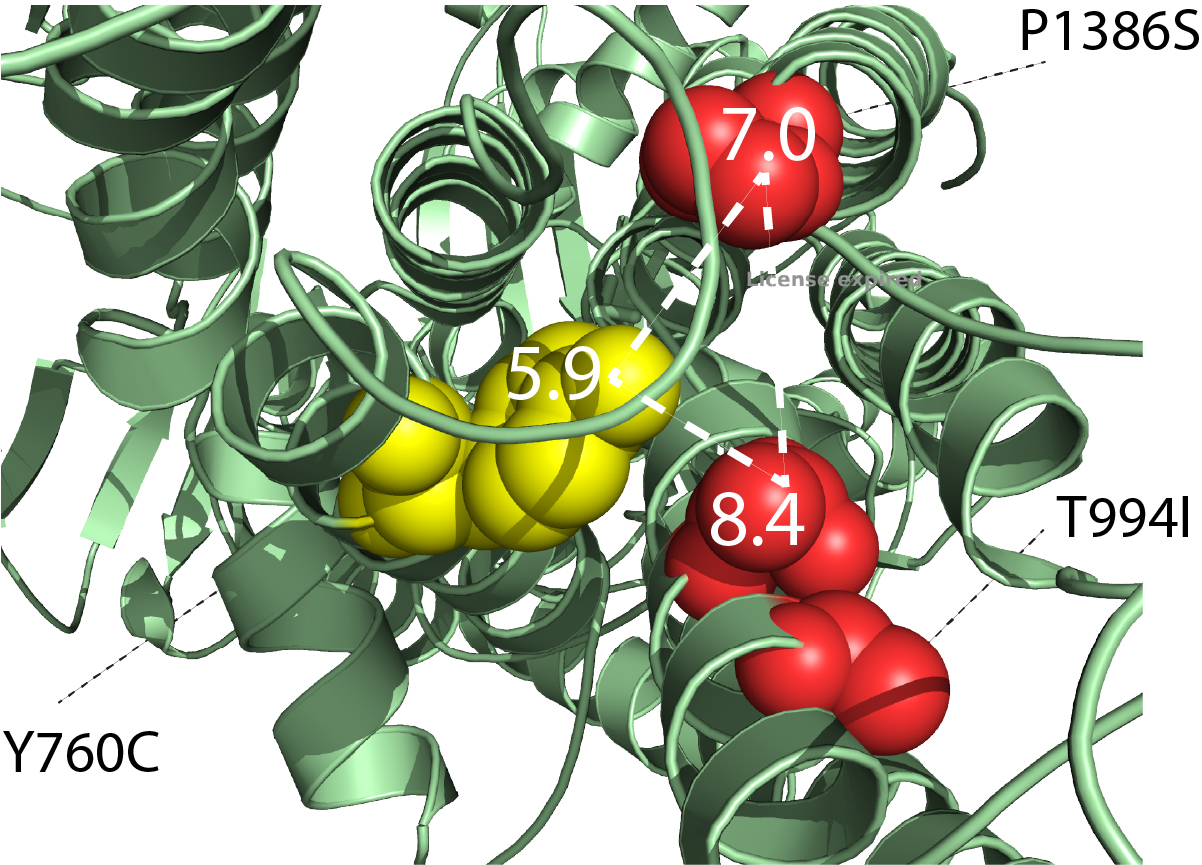

Supplement: Supplementary_materials_ddag061 [file supplementary_materials_ddag061.zip › ATP7A_VUS_SupplemFig6_Revision2.jpg]
